# Nimotuzumab-cisplatin-radiation versus cisplatin-radiation in HPV negative oropharyngeal cancer

**DOI:** 10.18632/oncotarget.27443

**Published:** 2020-01-28

**Authors:** Vanita Noronha, Vijay Maruti Patil, Amit Joshi, Manoj Mahimkar, Usha Patel, Manish Kumar Pandey, Arun Chandrasekharan, Hollis Dsouza, Atanu Bhattacharjee, Abhishek Mahajan, Nilesh Sabale, Jai Prakash Agarwal, Sarbani Ghosh-Laskar, Ashwini Budrukkar, Anil K. D’Cruz, Pankaj Chaturvedi, Prathamesh S. Pai, Devendra Chaukar, Sudhir Nair, Shivakumar Thiagarajan, Shripad Banavali, Kumar Prabhash

**Affiliations:** ^1^Department of Medical Oncology, Tata Memorial Hospital, HBNI, Mumbai, India; ^2^Mahimkar Laboratory, Cancer Research Institute (CRI), Advanced Centre for Treatment Research and Education in Cancer (ACTREC), Tata Memorial Centre (TMC), HBNI, Navi Mumbai, India; ^3^Section of Biostatistics, Department of Epidemiology, Advanced Centre for Treatment, Research and Education in Cancer, Navi Mumbai, India; ^4^Department of Radiation Oncology, Tata Memorial Hospital, HBNI, Mumbai, India; ^5^Department of Head and Neck Surgery, Tata Memorial Hospital, HBNI, Mumbai, India; ^*^These authors contributed equally to this work

**Keywords:** HPV negative, oropharynx, nimotuzumab, weekly, cisplatin

## Abstract

Background: Addition of nimotuzumab to weekly cisplatin and radiation improves outcomes in head and neck cancer. HPV negative oropharyngeal cancer has unsatisfactory treatment outcomes and is a candidate for escalation of treatment. We wanted to determine whether the addition of nimotuzumab to cisplatin-radiation could improve outcomes in these poor-risk tumors.

Methods: This was a subgroup analysis of a phase 3 randomized study. In this study, locally advanced head and neck cancer patients undergoing definitive chemoradiation were randomly allocated to weekly cisplatin (30 mg/m2 IV)- radiation (66–70 Gy) {CRT arm} or nimotuzumab (200 mg weekly) -weekly cisplatin (30 mg/m2)-radiation (66–70 Gy) {NCRT arm}. The data of HPV negative oropharyngeal cancer was extracted from the database of this study for the analysis. HPV testing was done with p16 immunohistochemistry (IHC) staining and reported according to the CAP criteria. The outcomes assessed were progression-free survival (PFS), disease-free survival (DFS), locoregional control, and overall survival (OS). Interaction test was performed between the study arms and HPV status prior to doing any HPV specific analysis for each of the studied outcomes. Kaplan Meier estimates for 2 year OS with 95%CI was calculated. The hazard ratio was obtained using COX regression analysis.

Results: We had 187 HPV negative oropharyngeal cancers, 91 in the CRT arm and 96 in NCRT arm. The interaction test was significant for PFS (*p* = 0.000), locoregional control (*p* = 0.007) and overall survival (*p* = 0.002) but not for DFS (*p* = 0.072). The 2- year PFS was 31.5% (95%CI 21.5–42) in CRT arm versus 57.2% (95%CI 45.8–67.1) in NCRT arm (HR -0.54; 95%CI 0.36–0.79, *p* = 0.002). The 2-year LRC was 41.4% (95%CI 29.8–52.6) in the CRT arm versus in 60.4% (95%CI 48.7–70.2) in the NCRT arm (HR -0.61; 95%CI 0.4–0.94, *p* = 0.024). The addition of nimotuzumab also lead to an improvement in 2-year OS from 39.0% (95%CI 28.4–49.6) to 57.6% (95%CI 46.3–67.4) (HR-0.63, 95%CI 0.43–0.92, *p* = 0.018).

Conclusions: The addition of nimotuzumab to weekly cisplatin-radiation improves outcomes inclusive of OS in HPV negative oropharyngeal cancers.

## INTRODUCTION

Locally advanced oropharyngeal cancers possess a unique challenge. The anatomical location along with multiple physiological functions associated with this structure makes open surgical resection a difficult and moribund procedure [1]. Hence in locally advanced oropharyngeal cancers, concurrent chemoradiation is used primarily for management. The prognosis of these tumors is heavily dependent on the Human Papilloma Virus (HPV) status [2]. Patients with HPV positive disease have a favorable prognosis as opposed to HPV negative disease [2]. The incidence of HPV positive disease is variable across the globe with the incidence in North America in the range of 48–81.4% [3], and 15–22.8% in the Indian subcontinent [4].

Efforts to improve outcomes in locally advanced oropharyngeal cancer have met with limited success [5, 6]. Recently a phase 3 randomized study was reported by us, in locally advanced head and neck cancers, testing the role of concurrent nimotuzumab in addition to weekly cisplatin and definitive radiation. The study met its primary endpoint of progression-free survival [7]. However, a similar study exploring the role of Cetuximab (RTOG 0522) was negative [8]. We had hypothesized that an improvement in outcomes in our study was seen due to the differential patient population in our study when compared to the RTOG 0552 study. We had a younger cohort of patients and a predominantly HPV negative disease [7]. This population, even in RTOG 0522 showed a trend towards improvement with the addition of cetuximab [8]. Another phase 3 study, exploring the role of cetuximab along with carboplatin -5 FU with radiation, reported improvement in outcomes with the addition of cetuximab [9]. The probable reason for this was probably due to the predominant HPV negative disease.

Taking this into consideration, we decided to perform a subgroup analysis of the HPV negative oropharyngeal cancer cohort, to study the absolute improvement in 2-year outcomes with the addition of nimotuzumab. We compared 2 year progression free survival (PFS), disease free survival (DFS), locoregional control (LRC) and overall survival (OS) between both arms.

## RESULTS

### Patient selection and baseline characteristics

We had 536 patients in the study, out of which 269 had primary in the oropharynx. P16 testing was feasible in 212 patients and 187 patients were p16 negative. These 187 patients are included in the current analysis. There were 91 patients in the cisplatin radiotherapy (CRT) arm and 97 in nimotuzumab-cisplatin radiation (NCRT) arm. The baseline characteristics were balanced between the 2 arms (Table 1).

**Table 1:** Baseline characteristics

Variable	Cisplatin-radiotherapy arm (*n* = 91)	Nimotuzumab-cisplatin-radiotherapy arm (*n* = 96)	*P*-value
Age, years			
Median age (range)	53 (31–75)	56 (34–70)	
Age > 60 years	23 (25.3)	30 (31.2)	0.272^*^
Gender			
Male	83 (91.2)	83 (86.5)	0.359
Female	8 (8.8)	13 (13.5)	
ECOG PS			
0	20 (22)	14 (14.6)	0.255
1	71 (78)	82 (85.4)	
Tobacco use			
Yes	85 (93.4)	88 (91.7)	0.783
No	6 (6.6)	8 (9.3)	
Subsite of malignancy			
Base of tongue	52 (57.1)	57 (59.4)	0.959
Tonsil	25 (27.5)	27 (28.1)	
Soft palate	10 (11)	9 (9.4)	
Posterior pharyngeal wall	4 (4.4)	3 (3.1)	
T category^†^			
T1–T2	25 (27.5)	16 (16.7)	0.08
T3–T4	66 (72.5)	80 (83.3)	
N category^†^			
N0–N1	35 (38.5)	35 (36.5)	0.88
N2–N3	56 (61.5)	61 (63.5)	
TNM Stage grouping^†^			
Stage III	14 (15.4)	18 (18.8)	0.794
Stage IVA	74 (81.3)	74 (77.1)	
Stage IVB	3 (3.3)	4 (4.2)	
Histological Grade			
Grade 1–2	68 (74.4)	66 (68.8)	0.418
Grade 3	23 (25.3)	30 (31.2)	

Data are presented as number (%) unless otherwise specified.^*^ *P*-value provided is for the comparison of age < 60 years versus age > or = 60 years between the 2 arms. ^†^The staging was done according to the AJCC-UICC 7th Edition.

### Outcomes

The interaction test for HPV status (positive & negative) was significant for PFS (*p* = 0.000), LRC (*p* = 0.007) and OS (*p* = 0.002) but not for DFS (*p* = 0.072), suggesting a differential impact of the addition of nimotuzumab with respect to HPV status.

### Progression-free survival

**Figure 1:**
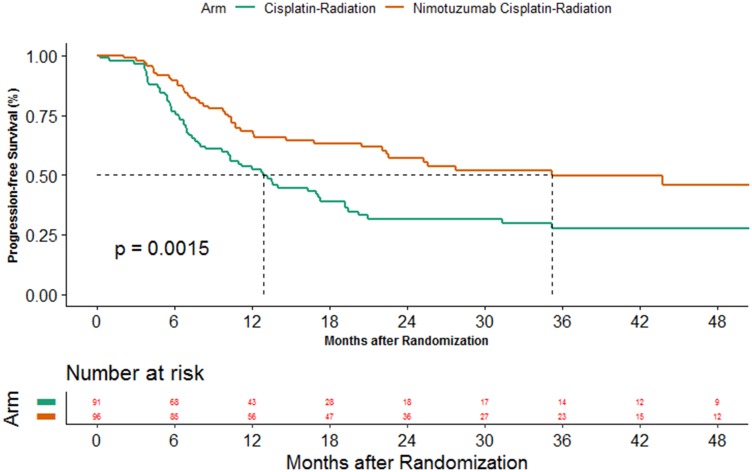
Kaplan Meier estimates of progression-free survival between the 2 arms.

At the time of data censoring 103 events had occurred, 60 in CRT arm and 43 in the NCRT arm. The median PFS was 12.9 months (95%CI 8.47–17.3) versus 35.3 months (95%CI 22.10-NA) (*P*-value = 0.0015) in the CRT and NCRT arm respectively. The 2 year PFS was 31.5% (95%CI 21.5–42) in CRT arm versus 57.2% (95%CI 45.8–67.1) in NCRT arm (Figure 1). The unadjusted hazard ratio was 0.53 (95%CI 0.36–0.79, *P*-value = 0.002). The multivariate analysis for PFS is shown in Table 2. The adjusted hazard ratio was 0.53 (95%CI 0.36–0.8, *P*-Value = 0.002). The results of sensitivity analysis performed for PFS using a composite endpoint of progression or death was in line with the above-mentioned analysis. The unadjusted and adjusted hazard ratios were 0.62 (95%CI 0.43–0.88, *P*-value = 0.008) and 0.62 (95%CI 0.43–0.89, *P*-value = 0.009), respectively. The site of failure was locoregional in 45 patients (49.5%), locoregional with distant failure in 3 patients (3.3%) and distant failure in 12 patients (13.2%) in CRT arm. The patients with corresponding sites of failure in the NCRT arm were 32 (33.3%), 6 (6.2%) and 5 (5.2%), respectively.

**Table 2:** Table depicting the result of multivariate cox regression analysis for progression-free survival and locoregional control

Variables	Variable type	Reference	Hazard ratio (HR)	95%CI of HR	*P*-value
Progression-free analysis					
Arm	Binary	Cisplatin arm	0.5352	0.3588–0.7983	0.00218^*^
Age	Binary	Below 60 years	0.7138	0.4573–1.1141	0.13776
Stage	Binary	Stage III	1.7770	0.9587–3.2937	0.06786
ECOG PS	Binary	ECOG PS 0	0.8501	0.5337–1.3541	0.49418
Grade	Binary	Grade 1–2	1.2734	0.8227–1.9710	0.27816
Subsite	Binary	Non base of tongue	1.0607	0.7106–1.5835	0.77303
Time to locoregional control					
Arm	Binary	Cisplatin arm	0.6311	0.4094–0.9728	0.0371^*^
Age	Binary	Below 60 years	0.7268	0.4453–1.1861	0.2016
Stage	Binary	Stage III	2.0823	1.0250–4.2303	0.0425^*^
ECOG PS	Binary	ECOG PS 0	0.6850	0.4203–1.1165	0.1290
Grade	Binary	Grade 1–2	1.1278	0.6967–1.8256	0.6245
Subsite	Binary	Non base of tongue	0.9464	0.6121–1.4631	0.8042

ECOG PS: Eastern Cooperative Oncology Group Performance Status. ^*^Statistically significant values.

### Locoregional control

**Figure 2:**
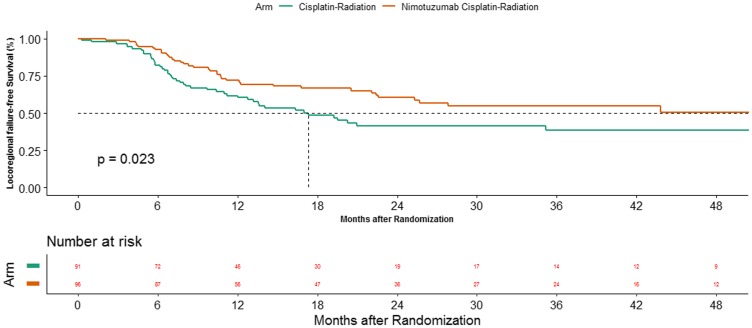
Kaplan Meier estimates of time to locoregional control between the 2 arms.

The improvement in PFS with the addition of nimotuzumab is largely contributed by an improvement in locoregional control. The median time to locoregional failure was 17.3 months (95%CI 12.0–56.3) and 60.3 months (95%CI 22.6-NA) in cisplatin arm and cisplatin-nimotuzumab arm respectively (*P*-value = 0.023). The 2-year LRC was 41.4% (95%CI 29.8–52.6) in CRT arm versus in 60.4% (95%CI 48.7–70.2) in NCRT arm (Figure 2). The unadjusted hazard ratio was 0.61 (95%CI 0.4–0.94, *P*-value = 0.024) while the adjusted hazard ratio was 0.63 (95%CI 0.41–0.97, *P*-value = 0.037). These results were confirmed in the sensitivity analysis. The unadjusted and adjusted hazard ratios were 0.68 (95%CI 0.48–0.99, *P*-value = 0.0358) and 0.69 (95%CI 0.43–0.89, *P*-value = 0.0494) in favor of the cisplatin-nimotuzumab arm, with the use of composite endpoint of locoregional failure or death.

### Overall survival

**Figure 3:**
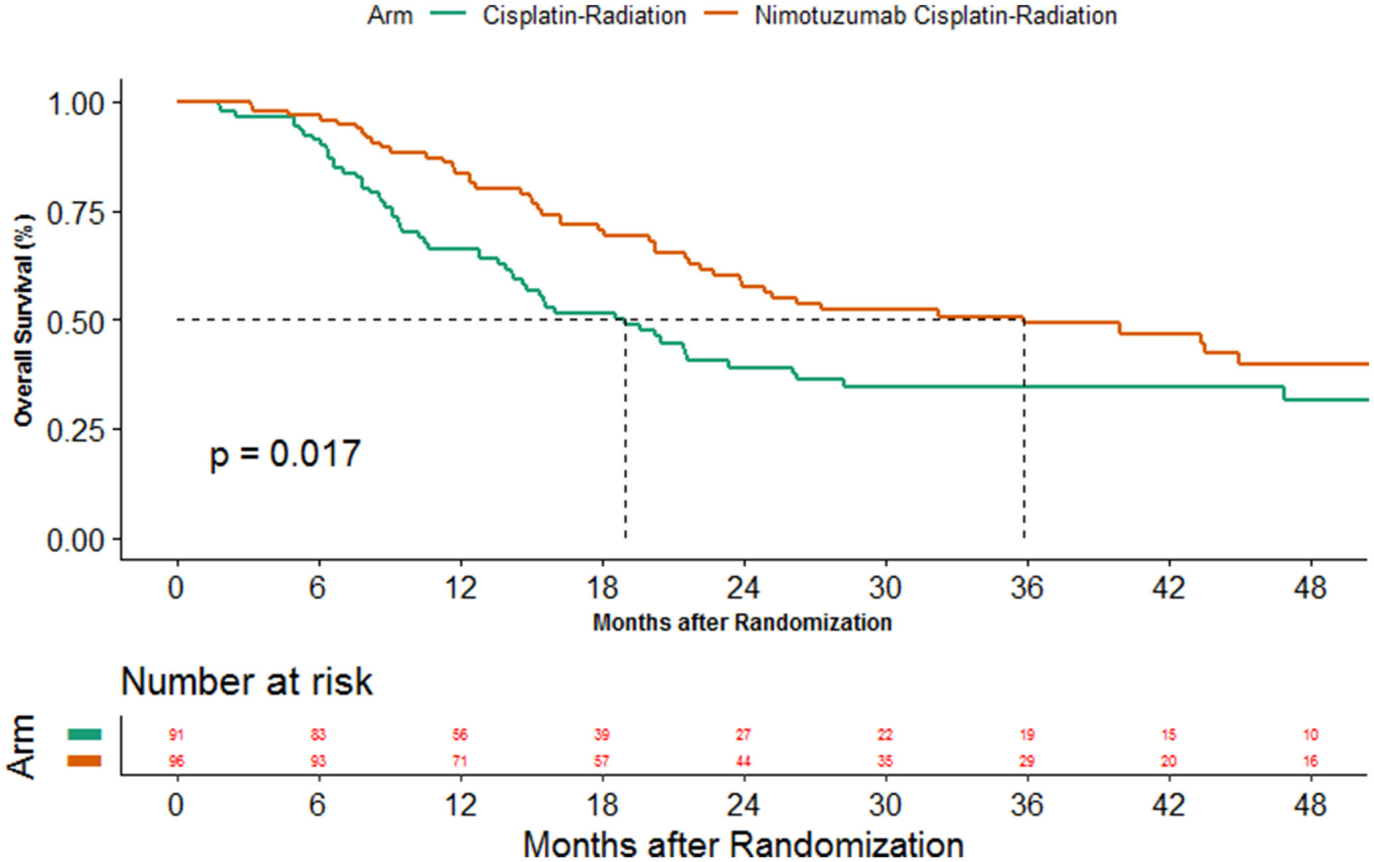
Kaplan Meier estimates overall survival between the 2 arms.

**Figure 4:**
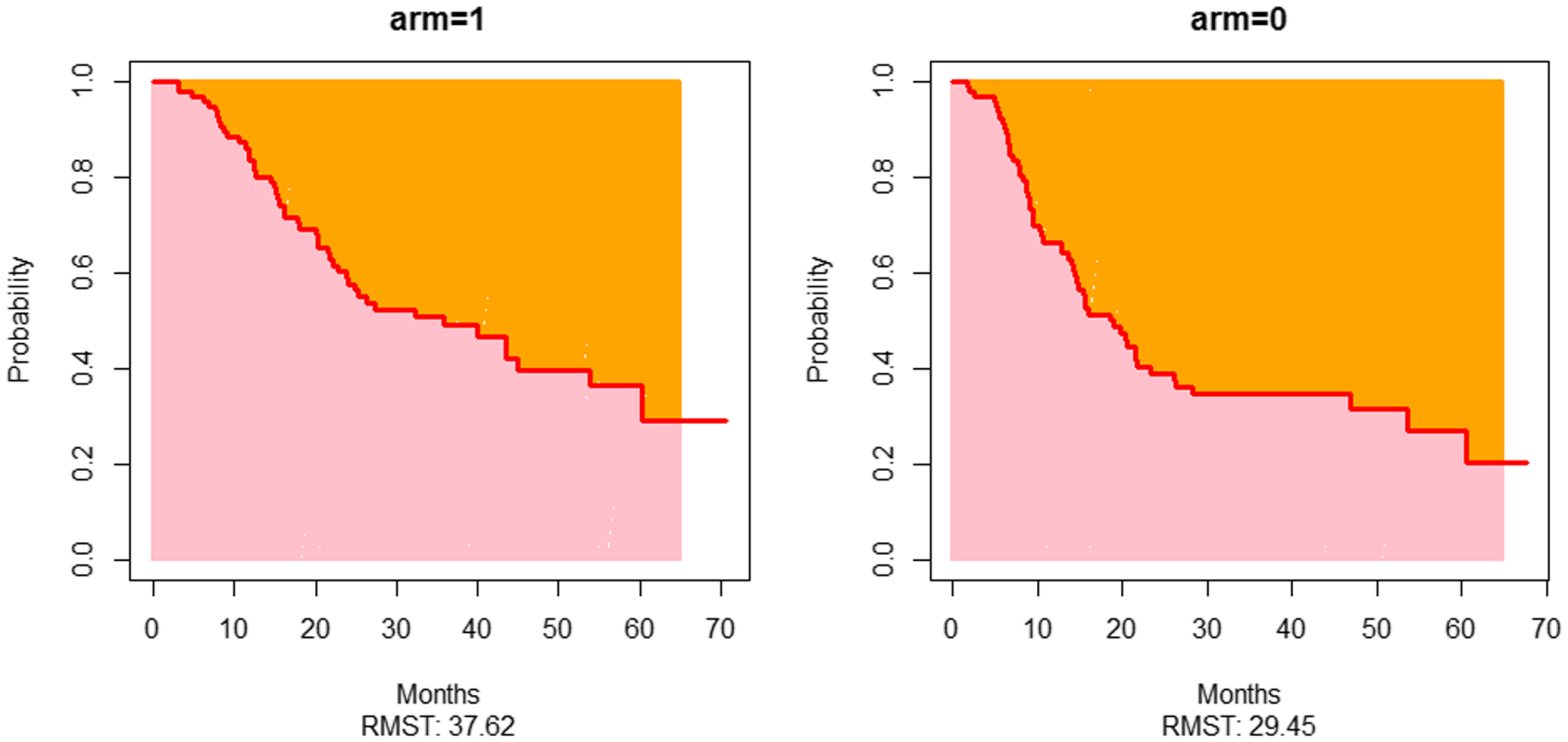
Restricted mean overall survival plots of both arms. arm = 0 represents the plot of the cisplatin radiotherapy arm while arm = 1 represents the plot of the Nimotuzumab-cisplatin radiotherapy arm.

The median follow up was 38.5 months (95%CI 36.2–46.3). At the data cutoff, 105 deaths had happened; 57 in cisplatin arm and 48 in the cisplatin-nimotuzumab arm. The median OS was 19.0 months (95%CI 14.2–23.4) in cisplatin arm while it was 35.9 months (95%CI 22.8–53.7) in the cisplatin-nimotuzumab arm (*P*-value = 0.017). The addition of nimotuzumab also led to an improvement in 2 year OS from 39.0% (95%CI 28.4–49.6) to 57.6% (95%CI 46.3–67.4) (Figure 3). The assumption for proportional hazard was violated (Supplementary Table 1) and hence, COX regression analysis was found to be an unsuitable method for comparison. Alternatively, restricted mean survivals were calculated for both arms and compared. The restricted mean survival estimated in cisplatin and cisplatin-nimotuzumab arm using data up to 65 months was 37.62 months (95%CI 32.5–42.74) and 29.45 months (95%CI 24.12–34.77), respectively (Figure 4), a difference of 8.17 months (95%CI 0.79–15.56; *P*-value = 0.03).

### Compliance to treatment

The data for treatment compliance and radiation technique are shown in Table 3. There was no difference in these factors between the 2 arms.

**Table 3:** Compliance data for radiation and cisplatin

Variable	Cisplatin-radiotherapy arm	Nimotuzumab-cisplatin- radiotherapy arm	*P*-value
	**(*n* = 91)**	**(*n* = 96)**	
Radiotherapy compliance			
Radiotherapy dose			
Median dose	70 (IQR 70–70)	70 (IQR 70–70)	–
100%	86 (94.5)	84 (87.5)	0.127
≥95%	86 (94.5)	85 (88.5)	0.193
Radiotherapy technique			
2-Dimensional	83 (91.2)	86 (89.6)	
IMRT	8 (8.8)	9 (9.4)	1.0
Not started	–	1 (1.0)	
Median package time in days	52 (IQR 49–55)	51 (IQR 49–54)	–
Treatment completed within 63 days			
Yes	88 (96.7)	93 (96.9)	1.0
No	3 (3.3)	3 (3.1)	
Gaps			
1 day or more	30 (33)	36 (37.5)	0.543
≥3 days cumulative duration	24 (26.4)	32 (33.3)	0.34
Systemic therapy compliance			
Cisplatin cycles			
Median	7 (IQR 7–7)	7 (IQR 7–7)	
7 or more	74 (81.3)	83 (86.5)	0.426
Cumulative dose 200 mg/m2 or above of cisplatin			0.858
Yes	72 (79.1)	77 (80.2)	
No	19 (20.9)	19 (19.8)	
Cisplatin dose reduction			
Yes	7 (7.7)	11 (11.5)	0.461
No	84 (92.3)	85 (88.5)	
Nimotuzumab	–		–
Median		7 (IQR 7–7)	

### Adverse events

The clinical adverse events were captured in 180 patients while laboratory adverse events were captured in 182 patients. The adverse events details between the 2 arms are shown in Supplementary Table 2.

## DISCUSSION

The results of the current study clarify the importance of treatment intensification in HPV negative oropharyngeal cancers. Locoregional control, progression-free survival and overall survival were improved with the addition of nimotuzumab to cisplatin and radiation. The absolute improvement in the 2 years LRC and PFS were around 20–25 percent. The corresponding improvement in OS was 18 percent. These improvements, in accordance with the ESMO magnitude of clinical benefit scale for curative treatment would classify as “A” [10].

As opposed to HPV related oropharyngeal cancer, HPV negative oropharyngeal cancers have worse prognosis. The median PFS, LRC, and OS in the current study are however, lower than reported in literature from the western world [8, 11, 12], [13–15]. The patient population in the current study had a history of tobacco use (90 percent) with the predominance of stage IV disease (80 percent), which probably resulted in lower survival. Both tobacco and stage IV are known bad prognostic factors [2, 16]. Another factor responsible for poorer outcomes would be the use of weekly cisplatin. However, it is unlikely as the cumulative dose of cisplatin received was 200 mg per M2 or more in nearly 3/4th of the patients. Dose intensification above 200 mg/m2 has questionable benefit [17]. These results in the control arm are similar to the results we previously reported [16].

Addition of nimotuzumab as a radiosensitizer to weekly cisplatin and addition of cetuximab to carboplatin 5-FU resulted in an improvement in outcomes over weekly cisplatin and carboplatin-5 FU in locally advanced head and neck cancers [7, 9]. However, a similar study of the addition of cetuximab to 3 weekly cisplatin was associated with negative results [8]. The probable reason for this discrepancy was the nature of the population in these 3 studies. In our study and the french study, the predominant population was HPV negative while, in the RTOG 0552, the predominant population was HPV positive. We feel that dose intensification would work if the population had poor prognostic factors like HPV negative. Hence, this analysis was performed to study whether addition of nimotuzumab would have larger incremental benefits in a HPV negative population. And indeed it leads to larger incremental benefits as opposed to the whole cohort Supplementary Table 3.

The current study is not without limitations. It is a subgroup analysis. However, an interaction test was performed prior to doing the subgroup analysis, to determine whether nimotuzumab had a differential impact or not. The study had used weekly cisplatin and not 3 weekly cisplatin, and so, the outcomes of the analysis are only applicable for weekly regimen.

## MATERIALS AND METHODS

### Patient population and design of the study

This was a phase 3 randomized superiority study conducted between 2012–2018. The study protocol was approved by the institutional ethics committee and the study was conducted in accordance with national (Indian Council of Medical Research) and international guidelines (Good Clinical Practice and Declaration of Helsinki) on human research. The detailed inclusion-exclusion criteria with study protocol are already published [7]. The study enrolled patients with locally advanced head and neck cancer who were planned for curative intent therapy, had normal organ functions, no uncontrolled comorbidities and ECOG PS 0–2. Patients with primary in salivary gland, nasopharynx or thyroid were excluded. For the current analysis, we selected locally advanced oropharyngeal cancer patients who were considered as p16 negative on immunohistochemistry.

### Intervention in both arms

The standard arm received radical radiation to a dose of 66–70 Gy with conventional fractionation over 6–7 weeks. Altered fractionation schedules were allowed in both arms if the Biologically equivalent dose (BED) was around 70Gy10. The chemosensitizer used was weekly cisplatin –30 mg/m2. The dose of 30 mg/m2 was selected as it was considered as standard at our centre and had been proven effective compared to radical radiation in a randomized study [18]. In the experimental arm, in addition to cisplatin and radiation, nimotuzumab was administered as a flat dose of 200 mg, over 1 hour in 0.9% normal saline without any premedication. Cisplatin in both arms was administered over 1 hour with adequate antiemetic cover and hydration.

### HPV testing

HPV testing was done using p16 IHC. The procedure for performing HPV testing has been already published in the literature [19]. Interpretation of p16 was done in accordance with American pathologist consensus guidelines. Both negative and positive controls were performed while performing p16 IHC for each batch for quality assurance.

### Study conduct

All patients, post consenting, underwent screening procedures, which were complete blood count, renal function tests, liver function test, viral serology, pure tone audiometry, dental examination, speech – swallowing function testing, nutritional status examination and evaluation by a social worker for compliance. All patients underwent an examination under anesthesia for mucosal disease assessment and a contrast enhanced computed tomography (CECT) neck with upper thorax for staging. In patients who had N2 nodes, the imaging used as positron emission tomography (PET). The radiation was administered either via conventional 2-D technique or 3-D technique. Use of intensity-modulated radiotherapy was permitted as per physician’s discretion. The patients were assessed each week during the treatment phase. Post-treatment completion at 8 weeks, patients were assessed in a joint clinic with a PET. Further follow up was according to the study protocol. The patients were followed up until death.

### Endpoint definition

The primary endpoint was PFS. It was defined as the time in months from randomization until progression. Progression was defined as per RECIST version 1.1. The secondary endpoints were LRC, DFS and OS. Locoregional control was defined as the time in months from randomization till loco-regional progression. Overall survival was defined as the time in months from randomization till death.

### Statistical analysis

Descriptive statistics were performed. Nominal and ordinal data between both arms were compared using Fischer’s test. An interaction test was performed between HPV status and outcomes (PFS, DFS, LRC, and OS). Only if the interaction test was positive (*P*-value of 0.05 or below), which suggested that the addition of nimotuzumab had a differential impact on outcome in accordance with HPV status, further analysis was done on HPV negative patients.

The subgroup had 187 patients. Considering a type 1 error of 5% and a type 2 of 20%, this subgroup was powered to rule out a 20% absolute improvement at 2 years in PFS, which was the primary endpoint.

Kaplan Meier method was used for estimation of the probability of PFS, LRC, and OS in each arm. The median estimates with the 95% confidence interval (CI) were reported. The 95%CI was calculated in accordance with Brookmeyer and Crowley method. The unadjusted hazard ratio (HR) and adjusted HR (adjusted for prognostic factors) with its 95%CI interval were calculated using the COX regression analysis with Efron’s method of tie handling, with CRT arm being considered as reference. The assumptions of proportional hazard model were checked using Schoenfeld residuals and assumptions were met for PFS and LRC. However, the assumption was violated for OS. Hence mean overall survival between the 2 arms were calculated using the restricted mean survival method. The difference between the restricted means between the 2 arms with its 95%CI were calculated. A *p*-value of 0.05 or below was considered as significant.

## CONCLUSIONS

The addition of nimotuzumab to weekly cisplatin-radiation improves outcomes inclusive of OS in HPV negative oropharyngeal cancers and this regimen should be considered as standard if 3 weekly cisplatin 100 mg/m2 regimen is not used.

## SUPPLEMENTARY MATERIALS


