# Supplementary material for: Nimotuzumab-cisplatin-radiation versus cisplatin-radiation in HPV negative oropharyngeal cancer
Source: Oncotarget. 2020 Jan 28;11(4):399–408. doi: 10.18632/oncotarget.27443 (PMC6996911; doi:10.18632/oncotarget.27443)
Supplement: Supplementary file 1 [file oncotarget-11-399-s001.pdf]

Nimotuzumab-cisplatin-radiation versus cisplatin-radiation in HPV negative oropharyngeal cancer

SUPPLEMENTARY MATERIALS

Supplementary Table 1: Table depicting the result of multivariate cox regression analysis for overall survival

Variables	Variable type	Reference	Hazard ratio (HR)	95% CI of HR	*P*-value	*P*-value for proportional hazard assumption
Arm	Binary	Cisplatin arm	0.6424	0.4354–0.9477	0.0257	0.0426
Age	Binary	Below 60 years	0.7735	0.5006–1.1951	0.2473	0.7478
Stage	Binary	Stage III	1.5857	0.8730–2.8802	0.1300	0.1898
ECOG PS	Binary	ECOG PS 0	1.0495	0.6542–1.6836	0.8412	0.8733
Grade	Binary	Grade 1–2	1.0483	0.6773–1.6224	0.8325	0.8437
Subsite	Binary	Non-base of the tongue	0.9383	0.6353–1.3859	0.7490	0.2250

The *p*-value in the last column depicts that the assumption of proportionality was violated.

Supplementary Table 2: Adverse events between the 2 arms

Variable	Cisplatin - radiotherapy arm		Nimotuzumab-cisplatin - Radiotherapy arm		*P*-value
	**All Grades**	**Grade 3–5**	**All Grades**	**Grade 3–5**	**Grade 3–5**
Hematological adverse events					
Anemia	71(80)	1(1.1)	78(83)	1(1.1)	1
Neutropenia	15(17)	3(3.4)	13(13.8)	–	0.111
Thrombocytopenia	20(22.7)	2(2.3)	16(17)	–	0.232
Biochemical adverse events					
Increased serum creatinine	10(11.4)	–	8(8.5)	1(1.1)	1
Increased AST	17(19.3)	2(2.3)	14(14.9)	–	0.232
Increased ALT	28(31.8)	2(2.3)	26(27.7)	–	0.232
Electrolyte disturbance					
Hyponatremia	83(94.3)	32(36.4)	81(86.2)	41(43.6)	0.365
Hypokalemia	3(3.4)	–	5(5.3)	–	–
Hypomagnesemia	28(31.8)	–	38(40.4)	–	–
Local radiation adverse events					
Mucositis	86(97.7)	53(60.2)	90(97.8)	66(71.7)	0.117
Radiation dermatitis	84(95.5)	27(30.7)	83(90.2)	25(27.2)	0.625
Odynophagia	86(97.7)	39(44.3)	91(98.9)	40(43.5)	1
Dysphagia	77(87.5)	26(29.5)	76(82.6)	32(34.8)	0.524
Gastrointestinal adverse events					
Nausea	47(53.4)	–	46(50)	–	–
Vomiting	25(28.4)	1(1.1)	20(21.7)	–	0.489
Weight loss	50(56.8)	–	57(62)	–	–

All adverse events are reported as number (%) unless otherwise specified. Acute adverse events - laboratory parameters were captured in patients for 182 patients (88 in Cisplatin - radiotherapy arm and 94 in Nimotuzumab-cisplatin - Radiotherapy arm) while non-laboratory parameters were available for 180 patients (88 in Cisplatin - radiotherapy arm and 92 in Nimotuzumab-cisplatin - Radiotherapy arm). ALT - Alanine aminotransferase. AST - Aspartate aminotransferase. SNHL - Sensorineural hearing loss.

Supplementary Table 3: Table depicting outcomes in the whole trial population excluding the human papilloma virus-positive oropharyngeal tumors (*n* = 24)

Variable	Cisplatin - radiotherapy arm (*n* = 254)	Nimotuzumab-cisplatin - Radiotherapy arm (*n* = 258)	Hazard ratio (95%CI)	*P*-value
2-year progression-free survival	48.1	62.1	0.649(0.499–0.845)	0.001
2-year locoregional control	55	68.1	0.615(0.459–0.823)	0.001
2-year overall survival	55.7	64.2	0.784(0.606–1.013)	0.062

Hence the sample size was 512.
